# Okweraliikirira and Okwenyamira: Idioms of Psychological Distress Among People Living with HIV in Rakai, Uganda

**DOI:** 10.21203/rs.3.rs-4656465/v1

**Published:** 2024-07-02

**Authors:** Nora S. West, Rosette Nakubulwa, Sarah M. Murray, William Ddaaki, Denis Mayambala, Neema Nakyanjo, Fred Nalugoda, Heidi E. Hutton, Pamela J. Surkan, Caitlin E. Kennedy

**Affiliations:** Department of Medicine, Division of Pulmonary, Critical Care, Allergy and Sleep Medicine; Rakai Health Sciences Program; Johns Hopkins Bloomberg School of Public Health, Department of Mental Health; Rakai Health Sciences Program; Rakai Health Sciences Program; Rakai Health Sciences Program; Rakai Health Sciences Program; Johns Hopkins School of Medicine, Division of Psychiatry and Behavioral Sciences; Johns Hopkins Bloomberg School of Public Health, Department of International Health; Johns Hopkins Bloomberg School of Public Health, Department of International Health

**Keywords:** Mental health, HIV, idioms of distress, Africa

## Abstract

**Introduction:**

Health and illness experiences are positioned within social and cultural contexts. Understanding the mental health and psychological distress of people living with HIV in highly affected communities is critical to addressing their needs and to ensure programming and interventions are targeted and appropriate.

**Methods:**

Grounded in the ethnomedical theoretical perspective, we conducted qualitative interviews to understand the experience and expression of psychological distress by people living with HIV in Rakai, Uganda. Participants included adults living with HIV (n=20), health workers (counselors, peer health workers, nurses, n=10), and key informants (n=12). Interviews were audio recorded, transcribed/translated, coded, and analyzed using thematic analysis.

**Results:**

Two idioms of distress, *okweraliikirira* (worry/apprehension) and okwenyamira (deep/manythoughts/lots of thoughts) were described as impacting people living with HIV. Both idioms were said to be alleviated by social support or counseling, but if left unaddressed could lead to more severe mental health problems and poor ART adherence.

**Conclusion:**

People living with HIV understand their psychological distress through culturally specific idioms; such distress can have deleterious impacts on well-being. Incorporating idioms of distress into screening and treatment for people living with HIV may improve identification of individuals in need and overall health services to address this need.

## Introduction

The World Health Organization and public health research community have called for increased attention to the mental health needs of people living with HIV in low and middle-income countries (LMICs) as part of strategic efforts to address the HIV epidemic, given the known impacts of poor mental health on HIV treatment engagement and outcomes (Collins, Holman, Freeman, & Patel, 2006; World Health Organization, 2008; Yotebieng et al., 2019). Despite these calls, there is limited contextualized research on the mental health needs of people living with HIV in low-income countries in Eastern and Southern Africa where HIV burden is high (Parcesepe et al., 2018) (Brandt, 2009), which limits action to holistically address wellbeing. Cross-cultural explorations of mental health suggest that the ways mental health and distress are understood and present differ across cultures (Chibanda, Cowan, Healy, Abas, & Lund, 2015; Summerfield, 2008; Tennyson, Kemp, & Rao, 2016). A systematic review of qualitative studies of depression experiences across 170 globally diverse populations found that while Diagnostic and Statistical Manual of Mental Disorders (DSM) criteria around which many mental health measurement instruments are structured were overwhelming present across the study populations, there were several recurring features of depression that are not part of current or previous versions of DSM criteria for depression, such as social isolation, excessive crying, anger, general pain, thinking too much or overthinking, heart issues, and headaches (Haroz et al., 2017).

Critiques of mental health assessments from both cross-cultural psychiatry and medical anthropology posit that culture plays a critical role in the study of mental health, yet the majority of research across diverse settings in Southern and Eastern Africa draws upon the assumption that terminology for or symptoms of mental health align with what is experienced by individuals in high-income settings (Bass, Bolton, & Murray, 2007; Kirmayer, 1989). Nichter applies the term “idioms of distress” to represent ways that individuals express and manifest distress in the context of personal and cultural meaning (Nichter, 1981). Idioms of distress may align with traits common in mental health disorders in high income countries (sadness, anxiousness, withdrawal from daily activities), but they may also be context and culturally specific behaviors or manifestations that are not represented or recognized on instruments developed in high income countries.

There is limited research exploring idioms of distress, including their causes and treatments, among people living with HIV in Africa. A study conducted in Uganda in the late 1990s found two idioms, *Yo’kwekyawa* (translated as “hating oneself”) and *Okwekubaziga* (translated as “pitying oneself”), described as part of mental health problems people experience related to HIV (Wilk & Bolton, 2002). Additionally, a systematic review exploring the use and applicability of the idiom of “thinking too much” found that when a specific illness was named as the cause of “thinking too much”, HIV/AIDS was reported in over 50% of included studies conducted in Africa and elsewhere (Kaiser et al., 2015). This review also demonstrated that while the idiom of “thinking too much” was described across many different cultures and contexts, its etiology, manifestation, impacts, and treatment approaches varied broadly (Kaiser et al., 2015). Taken together, this body of research suggests that exploring idioms of distress specifically related to the experiences of people living with HIV is warranted.

HIV prevalence in Uganda is 5.9% nationally (UNAIDS, 2017) but rates are significantly higher in the predominantly rural Rakai region, where median prevalence ranges from 14% in agrarian communities to 42% in fishing communities (Chang et al., 2016; Grabowski et al., 2014). There remains a pressing need to both effectively manage the health of people already living with HIV and prevent new infections in the Rakai setting.

For people living with HIV in rural Uganda, the experience and manifestation of psychological distress is likely uniquely shaped by their social and cultural context. A systematic review that looked at the integration of idioms of distress and cultural concepts of distress into mental health assessments found that incorporating idioms strengthened the validity of the measures or assessments, (Cork, Kaiser, & White, 2019) demonstrating the importance of exploring distress within cultures and groups before conducting mental health research or screening. In light of this research, we sought to elicit idioms of distress among people living with HIV in Rakai, Uganda. We utilized qualitative research methods to explore how psychological distress presents and is experienced by people living with HIV in Rakai. The overall aim of the study was to understand local conceptions of the types of mental health problems and idioms of distress that are most likely to be experienced by people living with HIV with the goal of identifying and/or adapting appropriate measures of mental health.

## Methods

### Study Setting and Population

From March 2020 to May 2021, we conducted semi-structured in-depth interviews (IDIs) and key informant interviews (KIIs) with people living with HIV, health workers, and key informants in Rakai, Uganda. For IDIs, adults living with HIV (ages 18–49) were recruited from participants in the Rakai Community Cohort Study (RCCS), an open ongoing population-based cohort study across 40 predominantly rural communities in the Rakai region, who had previously agreed to be recontacted for future studies.(Chang et al., 2016) We purposefully selected prior RCCS participants for our study to achieve balance in time since HIV diagnosis (≤ 18 months or > 18 months ago) and gender. The time period of less than or greater than 18 months was used based on the available data from the RCCS and to achieve adequate sample size of people more recently diagnosed with HIV. These two factors were chosen because they were thought to influence the perceptions on the experience of mental health among people living with HIV. IDIs were also conducted with health workers who regularly provide care to people living with HIV and who were employed by any of the three Rakai Health Sciences Program (RHSP)-affiliated clinics in Kalisizo, Kibaale, or Kasensero. Health workers were selected based on recommendations from RHSP staff for their knowledge and experience in mental health and/or experience caring for people living with HIV. All prior RCCS participants and health worker IDI participants were asked to recommend specific individuals in the community who they considered particularly knowledgeable about mental health for the KIIs. From participants who were willing to share contact details, a list of potential key informants was compiled. These key informants were contacted for interviews after healthcare provider and RCCS participant interviews were completed.

### Theoretical perspective

This research was guided by the ethnomedical theoretical perspective. At the crux of the ethnomedical theoretical perspective is the idea that health, illness, and individuals’ perceptions of health and illness are situated within the social and cultural context (Kleinman, 1978). As such, the terms that individuals use to describe illness, their explanatory models of illness causation and manifestations of illness, and the behaviors individuals perform related to care seeking are likely to differ across cultures. Within the ethnomedical theoretical perspective is ethnopsychology, which further seeks to understand how individuals within a cultural group view emotion and the self, and how they interpret and rationalize lived experiences (White, 1993). Also within the ethnomedical theoretical perspective is ethnophysiology, which describes cultural understandings of the functioning of the mind and body (Hinton & Hinton, 2002). The ethnomedical theoretical perspective guides both the elicitation of local terms, practices, and models for mental health and the relevant relationships between mind and body, as well as the idea that findings from research drawing upon this theoretical perspective can be utilized to strengthen future health research and programming (Kleinman, 1978).

### Procedures

Prior to conducting interviews, members of the Luganda-speaking study team, in consultation with study investigators, reviewed and discussed translations of the interview guides and specifically terms intended to elicit mental health models and distress and terms directly related to mental health. Final translations were reached through agreement by the Luganda-speaking team, with input on preserving intended meaning from study investigators. The majority of IDIs were conducted in Luganda by experienced RHSP qualitative data collectors: R.N., D.M., who received additional training relevant to this study. Four IDIs with health workers were conducted in English by N.S.W., who has experience conducting qualitative interviewing. All KIIs were conducted by R.N. and D.M. in Luganda. All participants provided informed consent. Four interviews were conducted in-person in March 2020, with all subsequent interviews carried out via telephone following the emergence of cases of COVID-19 in Uganda.

For health workers and individuals living with HIV, separate semi-structured interview guides were developed that explored mental health problems that occur among people living with HIV, how mental health problems impact people living with HIV, and how people seek care and treatment for these mental health problems. Specific terms for mental distress or disorder (e.g. depression, anxiety) were not used in these interview guides to allow for emergent concepts. KIIs also followed a semi-structured interview guide that asked questions about the most discussed mental health problems identified in the IDIs, including symptoms, perceived causes, sources of treatment, and impacts on HIV care. All participants were asked about the experiences of people living with HIV in their community, and not about their own personal experiences. All interviews were audio recorded. Interviewers completed debrief memos immediately following each interview, which were reviewed and discussed at study meetings. Completed interviews were transcribed and translated to English as necessary.

### Analysis

Transcripts of IDIs and KIIs were coded in ATLAS.ti software using thematic analysis (S, 2016). N.S.W. first analyzed IDI transcripts through an initial process of familiarization with the data, generation and application of codes, and finally identification and refinement of themes (Braun & Clarke, 2006). Analysis occurred in multiple rounds as data were collected and codes and themes added and refined. KIIs, which focused on the most discussed types of mental health problems or psychological distress identified in the IDIs, were analyzed through the same process described above. Symptoms, causes, impacts, treatments, and idioms of distress elicited from the interviews were also placed in a framework. Thematic maps, which serve as visual representations of codes and themes (Braun & Clarke, 2006), were constructed and refined during the analysis process to aid in identifying how manifestations of psychological distress or specific idioms of distress and their causes clustered together. Findings were compared across participant groups (e.g. people living with HIV, health worker, key informants), and for participants who were people living with HIV by time since diagnosis − ≤18 months ago or > 18 months ago. Findings were also compared by gender. Interpretations were shared with the data collection team and study investigators throughout the analysis process for input, feedback, and refinement of themes. Translations of terms from Luganda to English, including when terms could be combined or were distinct, were regularly discussed among the data collection team and study investigators.

The study was approved by the Institutional Review Boards of the Johns Hopkins Bloomberg School of Public Health, The Research and Ethics Committee of the Ugandan Virus Research Institute, and the Uganda National Council on Science and Technology.

## Results

The sample included 20 people living with HIV (n = 11 diagnosed ≤ 18 months ago, n = 9 diagnosed > 18 months ago), 10 health workers (n = 5 nurses, n = 3 HIV counsellors, n = 2 peer health workers), and 12 key informants, who included health workers, community leaders, and other community members. Participants who were more recently diagnosed with HIV (≤ 18 months) were more likely to raise stigma as a driver of experiencing psychological distress. There were no meaningful differences in participant responses by gender. We describe below when these factors (participant category, time since diagnosis) seemed to influence participant descriptions of mental health.

Two different idioms of distress were most discussed and prioritized by participants, and thus explored in greater detail in KIIs, were *okweraliikirira* (worry/apprehension) and *okwenyamira* (deep/manythoughts/lots of thoughts). Other mentioned, but not as commonly discussed or prioritized idioms included *okwekyawa* (self-hatred), *kumukyankalanya* (confused/disorganized mind), and *okwekubagiza* (self-pity). These idioms were sometimes discussed as independent mental health issues, and sometimes as signs or symptoms of other types of physiological distress.

### Okweraliikirira (worry/apprehension)

People in the Rakai region experience many different types of *okweraliikirira* (worry/apprehension). While *okweraliikirira* was said to potentially impact anyone, people living with HIV were noted to be more likely to experience it due to their HIV status and the associated factors that might cause additional distress. Fear of death from HIV, concerns about having to disclose one’s HIV status, anticipated or enacted stigma, and financial stressors due to HIV were drivers of *okweraliikirira* for people living with HIV, as described by a key informant:

A person experiences the problem of *okweraliikirira* first when told they contracted HIV [for the] first time; if they are a parent, they first *okweraliikirira* about whether they will live to raise their children. Second, if they are employed, they *okweraliikirira* that they will be chased from the job. Third, if they are married and tested alone, they *okweraliikirira* about divorce caused due to the partner seeing the [HIV] drugs.

Participants said that it was possible to tell if someone was experiencing *okweraliikirira* if they were sad and struggled to focus, as described by one participant, age 32, diagnosed with HIV > 18 months ago:

You may be having a conversation with your friend, but when he is not present, his thoughts are far, and he asks you, ‘What were you saying?’. There, he is *okweraliikirira* in his heart, he is not present even what you are conversing about it. He is not taking it seriously; he is only thinking about his condition [HIV status].

Additional ways that participants described being able to tell someone was experiencing *okweraliikirira* were if they had feelings of self-hatred or withdrew themselves from social situations.

Most participants who discussed *okweraliikirira* said that experiencing this issue could particularly affect adherence to ART, making it challenging for people living with HIV to either remember or focus on taking their HIV medication. Some participants said that the impact of *okweraliikirira* on adherence was cyclical, with both having to take medication and struggling with adherence causing individuals to experience *okweraliikirira.* Some participants said that men might also engage in risky sexual behavior when experiencing *okweraliikirira*. Aside from the impact on ART adherence, many participants said *okweraliikirira* could also negatively impact motivation to work, as described by one participant, age 40, diagnosed with HIV > 18 months ago: “They [people living with HIV] worry and stop working completely. They have no strength to grow food for the children. They have no strength to do casual labor to get food for the children who are about six and decide to give up.”

### Okwenyamira (deep thoughts, being in thoughts, many thoughts)

The idiom *okwenyamira* (deep or many thoughts) was described as more severe, and by some participants as a progression from *okweraliikirira*. Overall *okwenyamira* had similar HIV-related drivers as *okweraliikirira*, including coping with an HIV diagnosis and managing HIV care, financial stressors (associated with managing HIV care and other economic factors) and HIV-related stigma. Many participants discussed stigma and its impact on experiencing *okwenyamira*, but rarely was stigma described as something that happened at the clinic because of health workers. Most stigmatization was said to occur within the community or in the household, as described by one participant, age 23, diagnosed with HIV ≤ 18 months ago:

For example, I am infected, and I have HIV. I know that I have HIV, but the different thing is that my husband does not have HIV. We were both tested at the hub [through the RCCS] and he was found HIV negative. He was tested again after a month and was HIV-negative. Now, the problems you may experience are that your husband insults/verbally abuses you and begins mistreating you. He keeps nagging you with offending words that make you feel sad and *okwenyamira.*

There were a number of commonalities that were said to occur when someone with HIV experienced *okwenyamira* and *okweraliikirira*, including withdrawal or isolating from others, inability to focus, feelings of sadness, and weight loss. Additional ways that participants described being able to tell someone was experiencing *okwenyamira* included hopelessness, disruptions to sleep, self-pity, and anger, as described by a key informant:

*Okwenyamira* can be visible to the people if one is experiencing it. [The person] does shy away from the public, talk to themselves, sometime become taciturn with a lot of anger even though no one has insulted them. For you might interpret it as anger, but yet they are experiencing severe over thinking.

The impact of *okwenyamira* was also described by most participants as affecting the ability to adhere to ART, as described by one participant, age 23, diagnosed with HIV > 18 months ago: “*They are in thoughts all the time. If you do not stop being in thoughts when on HIV treatment, I think you reach a time and fail to take ARVs*.” Beyond adherence, participants also noted motivation or ability to work could be impacted along with the ability to complete daily tasks like household chores or tending to the garden, as described by a participant, age 40, diagnosed with HIV > 18 months ago: “It is still having *okwenyamira* that makes daily activities harder; you may be thinking about fifteen issues at the same time. If you are so stressed with deep thoughts, you may fall sick or break down.”. Some participants said that men were more impacted by *okwenyamira* because they had more financial responsibilities to family members and dependents, which caused them added distress that women did not always experience.

### Prevention, progression to severe disorder, and treatment

For both *okweraliikirira* and *okwenyamira*, participants described different factors that might make someone living with HIV more likely to experience the problem than others. Some participants described the ability to accept and cope with one’s HIV status as ways to prevent experiencing *okweraliikirira* and *okwenyamira*. Social support, often through disclosure of HIV status to household members or trusted friends, was also mentioned by many participants both as a potential way to help if one was experiencing mental distress in addition to being a protective factor, as described by a key informant:

A person of such kind usually experiences this problem of *okweraliikirira* because they have no one to talk to. They think about something their own way. Those that do not experience this problem of deep thoughts do have friends/colleagues who they shared or disclosed to about their health. Even if they ran out medicine, they can ask a friend for some medicine in case they are on the same treatment or can ask for transport in case they do not have money for transport to go to the clinic for treatment or does not have fast means of transport to the clinic and a someone helps them to quickly reach the facility because they disclosed to him/her and they know about his health problem. So, people of such kind rarely experience this problem of *okweraliikirira*.

Suicidal ideation or attempt were described in relation to both *okwenyamira* and *okweraliikirira*. With *okwenyamira*, suicidality was often talked about by participants as a downstream impact of worrying to the point that an individual might stop taking ART, which participants felt meant that they wanted to die and were suicidal. For *okweraliikirira*, suicide was more often described as an impact of not being able to alleviate or address *okweraliikirira* itself.

Depression was a mental disorder discussed regularly by health workers in interviews, and key informants were asked directly about it as it had been raised by health workers in initial interviews. The term/concept of “depression” was raised independently only by one participant living with HIV. There is not a singular term for “depression” in the Luganda language (the concept translates to *okukosebwa mubilowoozo okuyitiridda notuuka nokwekyaawa*), and for some key informants who were not in the healthcare field it was not a clear concept. Depression was often described by key informants as something that people living with HIV might progress to if they experienced *okweraliikirira* and/or *okwenyamira*, but were not able to overcome these issues or obtain care, as described by a key informant: “There are people who get fed-up with being in deep thoughts. They have no solutions. You find they have destroyed [their] things. Some reach an extent of committing suicide because they were diagnosed with HIV. Now where is hope if she is thinking of taking poison?”. While the term “depression” was rarely used by participants who were not health workers or key informants, the idea that someone living with HIV might progress from less severe distress to a more severe distress, often described as madness [*kulaluka*], was present in a number of interviews.

Counselling was mentioned by almost all participants as a way to treat or address both *okweraliikirira* and *okwenyamira*, and more severe mental disorders like depression or madness. This counselling was often described as facilitated through health facilities (either directly at the facility or through community-based peer health workers), though counselling from trusted family members or friends was also noted, as described by a participant, age 40, diagnosed with HIV ≤ 18 months ago:

You can meet a health worker and he or she counsels you. If that is not possible, you can get someone you trust -- you share with him or her your problems and he gives you advice. That is the medicine I think you can use for that problem. If it is possible to get a health worker or counselor and they give you counseling. In this case, they will give you knowledge on how to handle that problem such that you completely stop experiencing it.

## Discussion

This qualitative study exploring mental health and the HIV-related context for experiencing psychological distress found two common idioms of distress experienced by people living with HIV in Rakai, Uganda: *okweraliikirira* (worry/apprehension) and *okwenyamira* (being in thoughts or deep/many thoughts). While both idioms shared common manifestations, participants generally felt they were distinct and followed a progression from less severe to more severe mental distress. Both HIV and non-HIV related factors played a role in these experiences for people living with HIV. Financial stressors were regularly discussed as a driver of experiencing poor mental health, due to both general economic hardships and added financial burdens related to living with HIV. Both forms of distress were described as more likely to be experienced by people living with HIV and as having negative impacts on adherence to ART. Treatments and mitigation for experiencing distress primarily focused on formal counselling and/or informal social support.

Idioms of distress that involve thinking, overthinking, or being consumed by thoughts are well documented in the literature across non-Western populations worldwide (Kaiser et al., 2015). Causes for experiencing this type of distress vary across the literature, but the most commonly cited reasons are difficulty or discord in social relationships, economic concerns, traumatic or adverse past events, and illness (Kaiser et al., 2015). Our findings among people living with HIV support this and demonstrate how people living with HIV may be particularly vulnerable to experiencing “deep or many thoughts” because of how HIV influences relationships, the role of stigma in the daily life of people living with HIV, increased economic pressures, and coping with a lifelong illness and treatment. For people living with HIV, the intersection of multiple drivers for experiencing this idiom of distress may be unique and thus warrants attention.

The extant literature on idioms of distress that involve thinking, overthinking, or being consumed by thoughts also shows overlaps with some common mental disorders, such as depression, anxiety, and post-traumatic stress disorder (Kaiser et al., 2015). Additionally, “thinking too much” has been found to be prominent in descriptions of depression specifically among people living with HIV (Mayston et al., 2020). While some have appropriately cautioned against using this idiom as proxy for a specific mental disorder given the differences in manifestation, lack of mapping to a single construct, and the sociocultural contexts in which it may impact people, there is likely utility in incorporating it in to screening, e.g. determining who may require further evaluation and psychosocial support (Kaiser et al., 2015). Use of screeners for idioms of distress may help identify and support people experiencing non-specific distress. Incorporation of idioms of distress into measures may be useful to track changes in wellness associated with psychosocial services. The Luganda adapted version of the Hopkins Symptom Checklist (HSCL) measure for depression does incorporate *okweraliikirira* as a term/symptom for the depression section of the checklist, and a translation of “thinking too much/too many thoughts” as part of a measure of quality of life (Bolton, 2001). To our knowledge this is the only measure of mental health that utilizes these idioms of distress in the Ugandan/Luganda speaking setting. Other commonly translated and used measures of depression like the PHQ-9 do not incorporate these idioms of distress and have also been shown to perform poorly when validated among Luganda-speaking individuals (Nakku et al., 2016). Aside from the adaptations made to the HSCL, in the Uganda setting and Luganda language-based research or practice, there are no known measures that utilize idioms directly for distress or anxiety, identifying a potentially significant gap in appropriately identifying individuals in need of programming and support.

It is worth noting that in this study only one participant who was not a health worker or participant with some sort of healthcare training independently raised “depression” as a problem that impacts people living with HIV. Interestingly, a recent qualitative synthesis of explanatory models of depression in Africa found that people living with HIV, and particularly those engaged in HIV care, were familiar with biomedical models for depression (Mayston et al., 2020). The authors of this synthesis suggest the reason may be engagement with the healthcare system where people living with HIV may be exposed the idea of depression as an illness and learn how it manifests. This finding is contrary to our data among people living with HIV in Rakai. Reasons for this could include our approach to data collection. We utilized questions that did not ask about any specific mental distress, disorder or construct, but rather tried to elicit local understandings of mental health and distress among people living with HIV without a presupposition of what those might be or how they might manifest. Further explanation could be that in this setting, there is limited incorporation of mental health screening and services into HIV programming, so people living with HIV may be less familiar with terms like depression and mental health than in other contexts. Regardless, the absence of depression from the non-biomedical cultural discourse around mental health demonstrates the importance of attention to idioms of distress in research and programming. Additionally, it has been suggested that using idioms of distress, as opposed to Western/Global North terms for common mental disorders, may often be less stigmatizing and allow individuals to both express and have their distress understood (Kaiser et al., 2015).

Interestingly, the idioms of distress most discussed in our study differed from the idioms of distress experienced by people living with HIV documented in a study conducted in the Rakai region more than 30 years earlier among the general population. In the former study, *o’kwekyawa* (translated as hating oneself) and *okwekubaziga* (translated as pitying oneself) were found to be among the most frequently reported problems experienced as a result of HIV (Wilk & Bolton, 2002). While both idioms were mentioned by some participants in our study, discussion of them was far less frequent than of *okweraliikirira* (worry/apprehension) and *okwenyamira* (being in thoughts or deep/many thoughts). One potential explanation for this is the evolution of the HIV epidemic. With universal access to lifelong ART and demonstrated efficacy of HIV treatment, it is possible that people living with HIV may experience different types of distress in the modern treatment era and understanding both how this distress manifests and how individuals talk about is important for both screening and intervention adaptation or development. Another possible explanation for differences between the two studies is the method of data collection. The former study used free-listing and key informant interviewing utilizing vignettes, whereas our study used open-ended, semi-structured interviewing. The former study also spoke to community members generally, and did not sample based on HIV status, which may have influenced the perception of the severity of issues experienced by people living with HIV at the height of the HIV epidemic.

Although experiencing poor mental health and psychological distress is not unique to people living with HIV, robust evidence from Uganda and other African countries shows that poor mental health can negatively impact engagement in HIV care, risky behavior, and adherence to ART (Croome, Ahluwalia, Hughes, & Abas, 2017; Kinyanda et al., 2018; Musisi et al., 2014; Nakimuli-Mpungu et al., 2012; Nakimuli-Mpungu et al., 2013; Wagner, Ghosh-Dastidar, Mukasa, & Linnemayr, 2020). Our findings add to this evidence base. Participants felt that experiencing idioms of distress may influence ART adherence and that distress can progress from mild to more severe, suggesting that screening for common idioms of distress may be particularly important. Furthermore, findings from this study align with existing evidence on the role that HIV-related stigma plays a role influencing poor mental health and distress among people living with HIV, particularly among individuals recently diagnosed with HIV (Bebell et al., 2021; Rueda et al., 2016). Experiencing HIV-related stigma in the healthcare setting has been both documented and addressed through intervention development and implementation (Feyissa, Lockwood, Woldie, & Munn, 2019). However, our findings indicated that this type of stigma was overwhelmingly not of concern to study participants. However, household and community-based stigma (both anticipated and enacted) were frequently discussed as a common and deleterious influence on mental health for people living with HIV. Interventions to address stigma at the community and household levels may be particularly challenging to develop and implement in low-resource settings, but our findings point to the importance of ongoing consideration of these types of interventions for the mental and physical wellbeing of people living with HIV.

This study is not without limitations. The first author and primary data analyst (X.X.X.) is not fluent in Luganda. However, Luganda-speaking team members co-developed the study design and were involved with all phases of the research, including discussions about definitions of idioms of distress and associated factors throughout data collection and analysis. Further, Luganda and English version transcripts of all interviews were maintained and re-read when analyzing whether terms/idioms were similar in meaning or distinct.

## Conclusion

In conclusion, idioms of distress that impact people living with HIV may be the result of a confluence of social and HIV-related factors that are specific to this population characterized by place, time, and language. Given the emphasis on the utility of counseling and social support on mitigating or treating distress, integration of these idioms into routine clinical care and mental health screening may help better identify individuals in need of additional support.
